# “You have to make it cool”: How heterosexual Black men in Toronto, Canada, conceptualize policy and programs to address HIV and promote health

**DOI:** 10.1371/journal.pone.0278600

**Published:** 2022-12-30

**Authors:** Roger Antabe, Kimberley Robinson, Winston Husbands, Desmond Miller, Andre Harriot, Kwesi Johnson, Josephine Pui-Hing Wong, Maurice Kwong-Lai Poon, John Wasikye Kirya, Carl James

**Affiliations:** 1 Department of Health and Society, University of Toronto Scarborough, Toronto, ON, Canada; 2 Graduate Department of Geography and Planning, University of Toronto, Toronto, ON, Canada; 3 Dalla Lana School of Public Health, University of Toronto, Toronto, ON, Canada; 4 Daphne Cockwell School of Nursing, Faculty of Community Services, Toronto Metropolitan University, Toronto, Ontario, Canada; 5 School of Child and Youth Care, Faculty of Community Services Toronto Metropolitan University, Toronto, Ontario, Canada; 6 School of Social Work, York University, Toronto, ON, Canada; 7 Toronto Public Health, Toronto, ON, Canada; 8 Faculty of Education, York University, Toronto, ON, Canada; Family Health International: FHI 360, UNITED STATES

## Abstract

**Background:**

Black Canadian communities are disproportionately impacted by HIV. To help address this challenge, we undertook research to engage heterosexual Black men in critical dialogue about resilience and vulnerability. They articulated the necessity of making health services ‘cool’.

**Methods:**

We draw on the analyses of focus groups and in-depth interviews with 69 self-identified heterosexual Black men and 12 service providers who took part in the 2016 Toronto arm of the weSpeak study to explore what it means to make health and HIV services ‘cool’ for heterosexual Black Canadian men.

**Results:**

Our findings revealed four themes on making health services cool: (1) health promotion as a function of Black family systems; (2) opportunities for healthy dialogue among peers through non-judgmental interactions; (3) partnering Black men in intervention design; and (4) strengthening institutional health literacy on Black men’s health.

**Conclusions:**

We discuss the implications of these findings for improving the health of Black Canadians.

## Introduction

COVID-19 has raised fundamental questions about whether or how Canada’s health care system addresses the needs of Black and other racialized communities [1, 2]. The disproportionate burden of COVID-19 cases among Black Canadians compounds the existing HIV epidemic in Black communities that has persisted over the last four decades [3]. Thus, Black Canadians’ experience of concurrent epidemics (i.e., HIV and COVID-19) underscores the precarity of their health and wellbeing. Furthermore, the realities of COVID-19 re-echo the failure of Canada’s health care policy regime to address Black people’s health needs as COVID-19 infections closely mirror that of HIV in Black communities [4, 5].

In Toronto for instance, 33% of COVID-19 cases in August 2020 were attributed to Blacks although they constitute only 9% of the city’s population [4]. Similarly, Black people in Canada accounted for over 25% of new HIV cases in 2019, though they represent about 4% of the country’s population [6]. By way of comparison, we note that white Canadians account for about half of Toronto’s population but only about a quarter of COVID-19 cases up to the end of mid-2021, and make up about two-thirds of the national population but less than one-third of new HIV cases in 2019. These staggering disparities problematize the suitability and utility of Canada’s approach to health interventions for Black communities [3].

Achieving health equity requires a carefully crafted approach to health promotion and community engagement. This approach foregrounds health policies to achieve equitable access to health system resources and equitable outcomes, and reorients health services to address structural conditions that undermine Black people’s health and wellbeing. For instance, when asked about how health services can be tailored to mitigate the HIV epidemic among Black Canadian men, one of the participants in our weSpeak research study (described in detail below) responded, “You have to make it cool.” This perspective suggests that policy makers are out of touch with the realities of accessing health service for Black Canadians. Furthermore, this call to “make it cool” references the urgent need to understand how the system is failing Black communities and how to make the system responsive to Black men’s needs and priorities. In this vein, we sought to explore what ‘cool’ means for health service utilization and health outcomes among self-identified heterosexual Black Canadian men in Toronto.

Scholars have emphasized that Black men’s vulnerability to HIV is driven in a major way by their marginalized positioning in the social determinants of health, which is a legacy of long standing structurally violent anti-Black racism [7–10]. From a critical race perspective, Black men’s insistence on ‘cool’ in health service provisioning directly questions the erasure of their history, environmental, economic, and political context in how health policy is framed, structured, and delivered in Canada. The failure has resulted in health interventions being based on stereotypic assumptions about the Black community and ways Black people experience their health.

In this paper, we examine the underlying meanings and implications of “You have to make it cool” for designing health information and services for self-identified heterosexual Black men living in Toronto, Canada. Our data are derived from the qualitative arm of the weSpeak research study in Toronto conducted in 2016. weSpeak was a 5-year mixed methods study to examine vulnerability and resilience to HIV among self-identified heterosexual Black men in four Canadian cities (Toronto, Ottawa, London and Windsor), and identify pathways to productively engage them in community responses to HIV. We expect that our analysis and discussion will help to frame and inform pandemic recovery, responses to HIV and other health-related disparities experienced by Black men and Black communities, and inform current debates about health equity. The weSpeak project used a community participatory approach to help understand and address their structural exposure to HIV. Furthermore, it sought to mobilize other health stakeholders such as local health units, AIDS service organizations (ASOs) and service providers in responding to the health needs of Black men. Among other issues, weSpeak participants discussed everyday challenges accessing social and health services, their lived experiences of HIV stereotypes and how the framing of health services and delivery work to keep them away from health services. They also discussed the potential reframing of health information as well as reorganization of health services delivery to synchronize with the sociocultural identities and values of Black communities and make such services more in tune with their needs.

### “Cool” and Black men’s health

‘Cool’ and ‘Cool Pose’ are related terms used in Black communities in North America to describe the way that Black men may present themselves through everyday social interactions. Cool pose allows Black men to “rescue their self-esteem and self-presentation” in a racist setting that is hard on the Black community (Davis, 2012; p. 535). The terms have come to be associated with how Black people respond to their daily lived realities of structural racism and how they ensure the wellbeing of self and culture [11].

Subsequently, the terms have evolved to shape how Black men and women socialize, react and relate to their environment in ways that ensure their preservation as a racial group [12–14]. Among young Black men, for example, acting cool or having a ‘cool pose’ has become a conduit for expressing their presence, building social networks, and gaining social currency within and outside the Black community. Also hinged on street credibility, cool ensures Black men are able to effortlessly navigate their surroundings and relate to others in a confident posturing [15]. For many Black men, cool serves as an indispensable medium to manage emotions in ways that preserve their lives in the presence of a biased law enforcement regime. It also ensures their dignity by masking their emotions and becoming detached and unrevealing in their daily interactions and activities [13, 15].

‘Cool’ also references Black men’s resistance to linear and conventional structures that reinforce the status quo and pin them to the bottom of the social and economic hierarchy. Thus, ‘cool’ signifies courage, confidence, mental fortitude, and difference in a racialized sociopolitical environment [13, 16]. Cool therefore responds to the unique social, cultural, economic and cultural circumstances of Black men relative to the White population [11, 12, 17]. The concept of ‘cool’ has also been linked to a certain level of ‘knowingness’ among Black men, hinged on unhindered access to privileged information. Accordingly, access to detailed and quality information as an attribute of ‘cool’ may help Black men safely navigate the ‘streets’ and become informed and prepared for impending situations that may adversely impact them [18]. In this regard, being ‘cool’ is to be receptive to information to ensure the survivability of Black men within their sociopolitical environments.

For Black men, making an act ‘cool’ creates an aura of difference, drawing attention to the plight of their community and also resisting conventional narratives about their challenges as a racial group [12, 19]. Therefore, for health information and interventions to be ‘cool’ and receptive to Black men, they must primarily be targeted at the needs of Black men by incorporating their lived realities and prioritizing the unique sociocultural identities of Black communities and Black men [20, 21]. The need to make health information ‘cool’ and acceptable among Black men and the larger Black community constitute a significant shift away from understanding Black men as “at risk.” Classifying Black men as “at risk” may absolve the state of its failure to centre their unique needs into the design of services. Indeed, labelling Black as “at risk” prioritizes blaming them for HIV, stereotyping their behaviours as necessarily deviant or anti-social, and requiring them to abandon a culture that mainstream health promotion interprets as hopelessly flawed [22]. Consequently, structuring health services to be ‘cool’ deviates from the stereotypical construction of Black men’s HIV risk to a new era of health care provisioning that employs the concepts, tools and media that resonate among the target population.

### Theoretical context

A critical social science paradigm underpinned the design of weSpeak, given the focus of our study on health equity and social justice for self-identified heterosexual Black men. This approach opens opportunities for understanding the complexities of society’s institutions and ideologies and unmasking unequal power relations steeped in multiple levels of inequality. Specifically, we draw on Critical Race Theory (CRT) and Intersectionality which are useful frameworks for examining how socially constructed identities define social relations and also impact access to critical resources including health services [23–25].

CRT problematizes racism as a system-wide or structural issue, where the hegemony of Whiteness is deeply embedded in institutions, laws, regulations, and societal practices [23, 26]. Relatedly, Intersectionality considers multiple and concurrently existing social identities, and how these shape each other to inform one’s social position as an experience of privilege and/or oppression [25]. In essence, it is a framework for understanding power relations in society inclusive of how this defines and shape the quality of life of individuals within the same social environment [25, 27].

In the context of the current HIV study, these two approaches are useful for understanding the social identities and experiences of Black men and how these shape their HIV vulnerability and resilience. For instance, the construct of hegemonic masculinity hinged on Whiteness tends to limit Black men’s ability to accrue and use the privileges that come with it [28, 29]. Thus despite Black men’s best efforts and notwithstanding their level of academic, social, political or economic achievement, the power that White-centered hegemonic masculinity offer remains elusive and beyond their grasp [28]. In a social structure that privileges whiteness, their identity as Black men limits their access to health services and increase their vulnerability to poor health outcomes [30].

Structural racism shapes access to important social resources including health. However, there has been a tendency to interpret Black men’s poor health outcomes out of context. For instance, their masculinity practices have been interpreted as dominated by adverse behaviors (such as unrestraint, multiple sexual partners and condomless sex) that reinforce racist stereotypes of Blackness [31]. These stereotypical interpretations are then invoked to explain Black men’s heightened exposure to HIV. In our current analysis, CRT and Intersectionality are useful for unpacking the existence of insidious and obvious forms of structural racism and how this sustains the marginalization of Black people, which culminates in their experiences of poor health outcomes. Together, these theories make explicit how the commonplaceness of racism in the lives of Black men makes them vulnerable to poor health outcomes, but at the same time how they use ‘cool’ to mobilize themselves and advocate for health delivery that addresses their needs [32, 33].

## Data and methods

While the weSpeak project as whole used a mixed methods approach to data collection, findings for this paper draws on only the qualitative component in Toronto. Findings are presented from both focus group discussions (FGs) and in-depth interviews (IDIs) with heterosexual Black men and service providers.

### Participant recruitment

In 2016, we recruited self-identified heterosexual Black men (i.e., who identified as African, Caribbean or Black) who lived in Toronto and communicated in English or French, using a snowballing framework that incorporated targeted sampling to ensure heterosexual Black men from diverse backgrounds captured for the study. To capture the spectrum of experiences of participants, the strategy was to recruit Black men with varying ages, settlement or immigration statuses, and socioeconomic backgrounds. A detailed description of the recruitment strategy for weSpeak participants in Toronto published elsewhere [34]. We distributed recruitment materials throughout the city, focusing in particular on spaces and events where Black men congregate. Some participants were also recruited through word-of-moth contact among their networks, or from information available on the study website. Overall, prospective participants contacted the project coordinator to be screened for their eligibility to take part in the study.

We also recruited service providers who were directly engaged in providing health or social services to Black communities. While race, gender and sexuality were not eligibility criteria for service providers to participate in the study, they should have been providing services directly to clients in their organization for a minimum of five years. This was useful in getting the perspectives of experienced service providers on the challenges and ways the Black community can be better served. In all, 69 heterosexual Black men 16 years or older and 12 service providers gave a written consent and participated in either a FG or IDI. The research ethics board at Ryerson University approved the study protocol.

### Focus groups and in-depth interviews

Focus groups were generally organized by age (16–24, 25–39, 40 and older), but we also organized one for French-Speaking participants (N = 6), and another for participants who self-identified as living with HIV (N = 10). This approach was intended to increase within group resonance, reduce within group power imbalances, and facilitate open discussion within the settings of the group [21, 35, 36].

Focus group discussions centered on understanding heterosexual Black men’s access to health services in general and HIV-related health services in particular. Participants discussed challenges, barriers and opportunities for accessing health services and how they understood their position as Black heterosexual men living in Canada. They further discussed the organization of health spaces and information and how it is connected (or not) to their history, socioeconomic positioning, and structural hierarchy. Similarly, among the focus group for service providers, discussions centered on their experiences serving Black men and ways health services could be structured and organized to be more meaningful to the Black community. Overall, in both FGs and IDIs, participants discussed ways should health information can be framed and disseminated to increase health service utilization among Black men. Prior to the start of each interview session, participants were informed pseudonyms would be used in research publications or public presentation of study findings

The in-depth interviews afforded participants the space and privacy to elaborate on their experiences accessing health services in Toronto. In addition, IDIs gave Black men the opportunity to elaborate extensively on how health services could be made ‘cool.’ Furthermore, the use of IDIs allowed Black men living with HIV the opportunity to participate in the study without the accidental disclosure of their HIV status within a group setting. Each participant received an honorarium of CAD $20.

### Data analysis

Research assistants audio-recorded and transcribed verbatim all FGs and IDIs, and transcripts were exported to NVivo for qualitative data analysis. Team members read through transcripts and identified emerging themes which were further discussed at the provincial team level. This process resulted in consistency among sites with some themes being merged, dropped or subsumed under existing themes. The coded transcripts were exchanged and discussed among team members to enhance intercoder reliability and resolve any disagreements.

We used a mixed inductive-deductive approach to data analysis. With this approach, the theme identification was partly influenced by our theoretical insights on how Black men are positioned to experience health and quality of health using the lenses of critical race theory and drawing on the social determinants of health framework. Drawing on critical interpretative approach for this perspective, we acknowledge how Black men’s access to and use of health and social services may be shaped by several intersecting structural factors including their history, sociopolitical context, and their social relations in the context of Canada. For instance, we were cognizant of the fact that the willingness of Black men to access health information is influenced by how these spaces are organized and the populations that serve as their primary target. The second part of our theme identification through the inductive approach was driven by the data [37, 38]. In the presentation of our findings, each quote is presented with the participant’s pseudonym, their age and if the quote is coming from a group or an in-depth interview.

## Results

Table 1 summarizes the profile of the 69 self-identified heterosexual Black men who participated in the focus groups (N = 53) and indepth interviews (N = 16). One noteworthy feature is that young men (16–24 years old) are well represented. Another is that participants were generally well educated (50% had post-secondary education), but reported low incomes (55% reported that they earned less than $20,000 annually). This apparent mismatch between education and income probably reflects the income position of the youth who participated (close to 40% of participants were aged 16–24), but is also consistent with the low-income position of Black Canadians [39].

**Table 1:** Socio-demographic profile of participants in Toronto focus groups and in-depth interviews (N = 69).

	N*	%
***Age group***		
16–24 years	25	39.1
25–44	18	28.1
45–64	18	28.1
65 and older	3	4.7
**Total**	**64**	**100.0**
***Education (highest level completed)***		
Less than high school	7	10.4
High school	26	39.4
Post-secondary**	33	50.0
**Total**	**66**	**100.0**
***Annual personal income ($)***		
Less than $20,000	36	55.4
20,000–39,999	9	13.8
40,000–59,999	10	15.4
60,000–79,999	3	4.6
80,000 and over	7	10.8
**Total**	**65**	**100.0**
***Primary ethno-racial identity***		
African	29	42.0
Black	14	20.3
Caribbean	26	37.7
**Total**	**69**	**100.0**
***Relationship status***		
Single	34	51.5
Married/common law	24	36.4
Other	8	12.1
**Total**	**67**	**100.0**
***Place of birth***		
Canada	35	53.0
Another country	31	47.0
**Total**	**66**	**100.0**
***Religious affiliation*** ***		
Christian	49	74.2
Muslim	10	15.2
**Total**	**66**	

*total does not always add to 69 because of missing data

**completed college or university

***participants could choose more than one response.

### Black cool, HIV and health service provision

Overall, participants felt disconnected from health promotion initiatives and approaches that did not prioritize their needs or tap their abilities. They offered their perspectives on health promotion that could help strengthen their health and wellbeing. Participants emphasized that health services can be made ‘cool’ if they are connected to their history, culture, and everyday lived experiences.

Findings emerged as four themes or narratives on making health services ‘cool’: (1) health promotion as a function of Black family systems; (2) opportunities for healthy dialogue among peers through non-judgmental interaction; (3) partnering heterosexual Black men in intervention design; and (4) strengthening institutional health literacy related to Black men’s health. In general, theme 1 addresses the needs of Black men as individuals, theme 2 considers how to enable networks of Black men to thrive, while themes 3 and 4 stress institutional change to acknowledge, accommodate and address health needs to achieve health equity. Below, we discuss and contextualize the themes from the interview narratives.

#### Health promotion as a function of Black family systems

Participants emphasized the Black family system as a unique place to target health information, which they premised on the important role the Black family plays in Black men’s socialization and community mobilization. They discussed how the ‘village’—representing the communal values imbued through the family system—has been depleted over the years. Still, the Black family represents a medium through which health information may be evaluated and disseminated among its members. In effect, designing health information for the Black family system may encompass structuring it to be more receptive to the values of Black family interactions. This way, Black men at an early age are socialized to critically engage with health information and services that are appropriate to their needs. A FG participant noted:

*I think it’s important for us to look in the family because there’s no more community*, *the village is gone*. *It really is*. *I think because I’m involved with so much youth programs*, *there’s a lot of youth programs out there in terms of fathers and stuff like that*. *The commitment is getting the young men to be involved in these programs*. [Delroy, FG, 35]

Some participants blamed the failure of health authorities to appropriately disseminate health information through the family system for some of the sexual and reproductive health challenges in the Black community. Excluding health information from Black family spaces does not only represent a missed opportunity to normalize discussion on sexual and reproductive health (especially among younger men), but may also imply a missed opportunity to normalize health-related discussions as an expression of personal cool:

*I’m noticing that a lot of us have come into this room and we didn’t really know each other*, *we’re strangers yet we’re having so much important conversations about sex and education*. *Part of me looks back and I think it’s a shame a lot of us probably don’t have these conversations with family members or siblings*. *It is because families try to avoid this conversation*. [Marvin, FG, 39]

Framing health information to specifically target Black families will position Black men to be more responsive and open to discuss health and access health services. Delroy, a 35-year-old FG participant reiterated: *“… we got to save ourselves*, *it starts with our family*. *We must raise young boys to be men*.*”*

#### Empowering dialogue and non-judgmental interactions among peers

Participants echoed that for health information to be ‘cool’ it must not only be sensitive to the needs and identities of Black heterosexual men, but it should also be delivered through healthy dialogue devoid of judgments and stereotypes about how Black heterosexual men become exposed to HIV or other health challenges. Black men hinted how their lived experiences may be misinterpreted or decontextualized to construct negative narratives about their HIV risk. Understanding the complex factors that shape their health experiences was key to making health services more relatable and relevant. In this regard, the framing and dissemination of health information and services must consider the structural conditions that predispose Black men to HIV infection and other poor health outcomes. To enhance cool as a societal value, social and health service spaces must accept difference. This will go a long way to reduce the judgement and blame some Black men experience in these spaces:

*If they could make those places and stuff [health information and services] relaxed and easy going*. *It is the worst to feel awkward in a place when you feel like you’re being judged*. *Make it more easygoing*. *Not like something you feel scared to talk about*. [Eaze, FG 16]

A cool society will support programs and services that are equitable and inclusive to Black men’s health needs. Moreover, the access points will allow Black men to safely and confidently access information and interact with service providers, especially with Black service providers:

*It’s about cultural sensitivity to Black men and their ego*. *It is about how we defend and present it*, *and how it interacts/intersects with their daily experiences*. *It is supposed to be a safe place where they can come and speak because the people there are Black…*.*we have to be able to build that trust that [Service provider for Black fathers] does*. [Tristan, FG-SP, 61]

Recognizing and addressing Black men’s lived realities as part of the commitments toward achieving health equity in Canada will mean reaching HBM in safe spaces and providing health information that are culturally safe and inclusive. For HBM, safe spaces include community places that are part of their everyday life. For instance, in a FG for Black men 25 years and older, a participant spoke about *“…taking it [i*.*e*. *HIV and health information] into places where it normally wouldn’t have been taken [by service providers] like barbershops and definitely churches”*. Many participants felt that in these everyday community spaces, Black men would be comfortable learning, discussing and evaluating information on sexual and reproductive health. Normalizing HIV information for Black men and communities would likely reduce stigma and fear that have hitherto fraught HIV information among Black men.

Some participants identified peer dialogue as a ‘cool’ tool for engaging Black men in these everyday spaces. Kadeem, a 25-year-old participant in an in-depth interview, felt that peer dialogue is a great conduit for building trust and HIV information dissemination *“Once you get them to open-up and show you certain things*, *then you can give them any information that they need*. *So*, *developing those [peer] relationships is good*.*”* As in other research [40], some participants identified the importance of integrating ‘fun’ into cool HIV prevention education, as Troy, a 28-year-old FG participant emphasized: *“That’s the only way that it really works*, *by mixing fun*. *The fun is getting it in through your brain without you even knowing it*.*”* For some participants, presenting HIV information in an innovative and non-conventional way

*…… is key so that it becomes cool to have that discussion and deal with that problem*, *and it’s not easy*, *because it’s years*, *generations*, *centuries of this perpetuation of this evil thing that goes on with us*. *So*, *we have to start chipping away with–with no immediate guarantee*, *but it’s work that needs to be done as part of that process*. [Tristan, FG-SP, 61]

In relation to HIV in particular, “cool” involves safe, healthy dialogue with and by Black men, devoid of stereotypical judgements about HIV risk and vulnerability, which reduces the associated stigma that creates a barrier for accessing HIV testing and treatment services.

#### Partnering HBM in intervention design and implementation

Participants emphasized the need for institutional cool in the delivery of health services. This requires an overhaul of institutional approaches to engaging Black men including building partnership with them in the design and dissemination of health services. For instance, engaging Black men to create and disseminate HIV and health information to peers can work to increase Black men’s attention, interest, and consumption of health information. They underscored the potential impact of Black men at the forefront of HIV information dissemination in their communities:

*Youth groups like us who are urbanized*, *have to go out there and talk to other youths and to help them to relate to us and open up their minds to the information we’re feeding them*. *Because if a person like you [i*.*e*. *non-Black male interviewer]*, *no offence*, *comes up to a group of people in the streets*, *like Black teens it’s not like they’re going to totally disrespect you*, *but they will not be interested in what you are saying*. *Whereas if a Black man like me goes up to a group of Black people and started talking about it*, *the conversation’s going to pick up and people are going to be interested and have their inputs*. [Jeron, FG, 25]

This means that Black men’s active involvement is integral to HIV and health information dissemination in health care spaces, community outreach programs, and health promotion activities in Black communities.

*……if you attack you got to attack full force*. *You can’t go in just half*. *It has to be full force*, *where everybody’s recognizing it*, *or majority of people are recognizing it…*..*In terms of promotion*, *say call a parade*, *having those events*, *just centering Black men around–putting Black men in the centre of awareness and focusing on them*. [Devonte, FG, 22]

Recruiting older Black men who are seen to embody ‘personal cool’ to lead in health promotion activities in Black communities will help to situate HIV and health information as ‘cool’ to younger Black men who look up to them as mentors. As Josh a 20-year-old-Black male observed in-depth interview, this strategy will help to normalize discussion of sexual and reproductive health “*…So that’s who they look up to and that’s who they want to aspire to be like*. *They change themselves; they start listening to them*, *they start watching them more*, *they start hanging out with people*, *they want to be like these guys* …” Further, Vince suggested that a pathway to institutional health literacy is through partnerships with popular, well known and accomplished Black men whose involvement in community engagement and dissemination activities will demystify HIV testing and treatment.

*Get the popular people to endorse it*. *If Drake endorsed it*, *he could get a million people to go get tested tomorrow*. *If you get tested*, *you get a free admission into a Drake concert*. *That place would be full*. *They can have a big music festival on AIDS awareness*, *where you have like 30*,*000 people*, *your admission is to get tested*. [Vince, IDI, 41]

Given the large following that accomplished individuals and community leaders attract, their platforms to discuss health within the Black community will help normalize such discussions particularly among younger cohorts of Black men who idolize them.

#### Strengthening institutional health literacy on Black men’s health

weSpeak participants reflected that they have been left out of the health policy environment and observed that their exclusion or marginalization has had adverse repercussions on how health institutions and experts understand Black men’s health. Some participants suggested that a new progressive turn in institutional health literacy is indispensable to positioning Canada’s health care to be responsive to Black men’s needs. Institutional health literacy constitutes a direct medium to achieving institutional cool as it requires changing the current deficit approach to an asset based one that works with the strengths and resilience of Black in HIV information dissemination.

*For Black men there’s certain drivers that make us tick and our resilience is directly related to those drivers*. *The things that make us seem or appear weak*, *we avoid*, *we stay away*. *It’s just like in marketing*, *if you’re selling a product*, *do you talk about its weaknesses or the strengths*? *You never talk about the weaknesses …*. *We have to make it cool to be honest and to be vulnerable for Black men*. [Tristan, FG, 61]

Ensuring institutional cool is also premised on the fact that existing frameworks have been deficient in unpacking Black men’s health needs in a holistic way. Thus, to achieve an in-depth perspective on the health experiences and health outcomes of Black communities, the existing approaches must be revised:

*It’s not saying that there aren’t those supports available*, *they’re there*, *but at the same time*, *there must be a certain level of depth and accountability from all fronts to make sure that it’s a holistic approach*. *So that Black men are informed and encouraged enough to know that this is where they need to be focused on in order to see the next day and not have to worry that they may come up with something because of the fact that they made the wrong decision*. [Adrian, FG-SP, 36]

A new approach to institutional health literacy will be more effective at garnering the appropriate attention and resources, and the involvement of all relevant institutions and groups:

*And it comes back to government*, *what are they doing to make sure people have the right education*? *What are they placing out there to make sure the percentage of STIs are going down*? *What are they focusing on*? *Are they focusing more on wars or more on society*? *… Realistically*, *the way I look at it now*, *they do much*, *but they don’t do as much as they should*. *They do very little because they are so focused on what they feel is other priorities*. [Marcus, FG, 23]

Though health institutions and health policy stakeholders may be reluctant to approve policy changes that deviate from the status quo, a more supportive interpretation of health literacy may be useful in eliminating health inequities:

*A lot of it comes in terms of advocacy on that systemic component*. *In the short amount of time that I’ve been working within the sector*, *I’ve had the opportunity to sit on advisory committees with prominent agencies that are supposed to be servicing Black men and in a lot of cases*, *major challenges that come into play is that there’s a resistance to actually appropriately and accurately label what the situation or issue is*. [Adrian, FG-SP, 36]

Black stakeholders may address this shortcoming by promoting critical understanding of health and wellbeing among Black communities. This will in turn help mobilize Black communities to strategically engage the mainstream policy experts. Addressing the structural factors that shape Black men’s health outcomes requires institutional cool where they are partnered in the design of health information.

## Discussion

Studies among Black men [9, 41] allude to structural barriers that impede their utilization of social and health services, as opposed to the need for culturally sensitive and structurally competent information on HIV and health. To position Black men to have unhindered access to health and HIV information that reduces their vulnerability and taps into their resilience against HIV, some Black men support the notion of informing health services and information with the notion of “cool”. Black men’s insistence on “making it cool” challenges health promotion practices in two important ways. First, Black men are challenging the structural inequity that underpins health promotion. Second, they are proposing that health services be restructured to achieve health equity. In this paper, we explored what it means to make health services and HIV health information cool for heterosexual Black men living in Toronto.

Our findings indicate that the present framework guiding health services delivery, including dissemination of HIV information and access to health services in Canada, may be neglecting the specific needs of Black men and the consideration of the contextual factors that shape their health experiences in Canada. To increase Black men’s use of health services and unhindered access to health information, social and health services must be redesigned and structured to straddle three levels of Black men’s cool. First, services and information for Black men must recognize them as individuals within family and other social networks that express their cultural identities. Second, health promotion efforts must be cognizant of the varied and unique experiences of Black men as a distinct constituency within the Canadian society. Third, institutional norms and practices must align with Black health needs. Specifically, this would require institutions to first deconstruct, co-create and transform narratives about how Black men experience poor health in Canada, and target them with specific interventions that are relevant and inclusive of their sociocultural contexts. Contextualizing this finding through the lenses of critical race theory, we have argued that Black men have been treated as the racial ‘other’ with a disregard for their distinct socio-cultural identities and lived experiences. This has culminated in their disconnection to health care services and spaces and subsequent over-representation in poor health outcomes in Canada [21]. In other words, structural racism forecloses beneficial access to health and social services [41].

To achieve health equity for their community, Black men call for the normalization of ‘cool’ within the larger Canadian society where their presence and health needs are not obfuscated in health services provisioning that prioritizes whiteness. There is urgency to their demand for recognition as a group or community with a legitimacy to thrive. A Canadian society that acknowledges ‘cool’ will enable them to live their normal lives without the usual barrage of stereotypes and social policing that work to create access barriers to social and health services.

The importance of the Black family as a medium through which health information can be targeted and normalized as ‘cool’ bestows on health policy makers, health institutions and practitioners responsibility to adapt their practices to Black family settings. Consistent with this observation, a previous study revealed that the Black family system is an indispensable source of fortitude, strength and resilience for heterosexual Black men as they navigate daily to reduce their structural exposure to HIV [42]. In the United States, Bowleg et al. [43] have also observed that the desire to protect family members from HIV served to motivate heterosexual Black men and underpinned their commitment to reducing their own exposure. In line with this evidence, targeting the Black family to make HIV and health information ‘cool’ has two prospective benefits for addressing HIV and poor health outcomes within the Black community. First, Black men will be socialized to discuss their health including sexual health at younger age that can normalize health discussions with other family members and professionals. Second, normalizing health discussions on sexual health and HIV provides a useful platform to reducing HIV stigma and increasing testing [44, 45].

Mostly drawing on behavioural perspectives, standard discourses on HIV exposure tend to emphasize individual level characteristics as key risk factors. Particularly for Black men who have been stereotyped as carriers of HIV, interpreting their HIV exposure as individual level choices offers limited opportunities for partnership in addressing their structural health vulnerabilities [7, 43]. As Resnicow et al. [20] argue, making HIV health information ‘cool’ and acceptable to Black men requires an in-depth engagement with Black men and synchronizing health information into both the ‘surface’ and ‘deep’ sociocultural structures. Black men also called for the creation of safe spaces to discuss and mobilize for their health. This is consistent with previous research among Black men who have sex with men that demonstrated the utility of safe spaces for providing social support, addressing stigma, as well as conducting HIV testing and campaigns [46].

The underrepresentation of Black men in the health care delivery cascade has worked to keep Black men as a population away from available services. ‘institutional cool’ represents a call to frame and structure social and health service delivery to prioritize Black men’s lived experience. Accordingly, increasing the representation of Black men in the frontlines of health information dissemination and health service delivery will help to connect the system to the Black community [47]. More generally, the infusion of messages on HIV and other health information into popular culture—created by Blacks to tell their story—will increase awareness and regularize discussions about health. For instance, a review of the rap/hip-hop lyrics on HIV found these mediums to be generally helpful in engaging young Black men and women in dialogue about HIV [48]. Further, Boutin-Foster et al. [49] have also emphasized the utility of reaching younger cohorts of Black men with HIV information through rap music in line with evidence that most Black youths consume rap/hip-hop music [50].

To help health policy stakeholders and institutions achieve their mandate of ensuring population health equity for structurally exposed populations, there is an urgent need for re-examining population health inequalities beyond biomedical descriptors. In echoing the findings from a previous study [51], it is critical for health institutions to have an in-depth knowledge and perspective on conditions that predispose Black men to poor health outcomes. This calls for the institutionalization of Black health literacy which will require a reconsideration of the training, structuring and resourcing of health institutions and practitioners to be informed about the conditions that shape Black health and impedes access to health care [21, 52, 53]. Improved Black health literacy within health institutions and among service providers, as participants suggest, will spearhead the discussion on community partnership in designing health interventions for Black men and the rest of the Black community.

Making health interventions cool signals an urgent need to re-structure these interventions through a two-pronged approach. First, the content of health information must be designed to be Black focused which requires the involvement of Black community members to help in adapting health messages and interventions to meet the needs of Black communities and Black men. Second, healthcare spaces need to be organized in a way that is welcoming of Black men and restructures how their needs and priorities are understood and addressed.

### Study limitation

Our study has some notable limitations. First, the study focused on only participants in Toronto and the GTA, therefore our findings may not necessarily capture the nuances of health care access for Black men more widely in Canada. Second, given that our study primarily focused on health care access for heterosexual Black men, our findings may not extend to other spheres of Black men’s social interactions including education and employment. Lastly, this paper reports on the qualitative component of the study, i.e., the experiences and perspectives of 69 heterosexual Black men and 12 service providers. Our aim is not to generalize the study results to represent the experiences of all heterosexual Black men in Toronto. Instead, we offer important contexts about what contributes to effective HIV services for HBM and communities. Despite these limitations, our findings make a substantial contribution to understanding Black health and how services could be reoriented as part of health promotion approaches to engaging Black men.

## Conclusion

Our findings on Black men’s interaction with health services in Ontario, and their expectations, have implications for achieving health equity in Canada. Making social and health services ‘cool’ is first and foremost to restructure and reorient health policy in Canada to acknowledge the role of structural barriers in Black men’s health experience. By calling for health policy and services to be ‘cool’ Black men are directly critiquing the status quo that guides the design and delivery of health services in Canada. Through this call, Black men question the assumptions and framework that underpin the design and delivery of social services. Presently the assumptions underpinning the health interventions in Black communities are devoid of equity considerations as they are detached from the realities of marginalized and structurally exposed populations in Canada. It is therefore quite paradoxical that despite Canada’s claim to cultural diversity and health equity, Black men’s worsening health challenges and risk of HIV have not been prioritized. Moving away from the predominantly biomedical framework as a guide to health service provisioning to one that incorporates the historical and sociocultural contexts of marginalized populations will be an important shift to achieve health equity for Black and other racialized groups.
